# The Schoolmaster or the Doctor?

**Published:** 1895-06-01

**Authors:** 


### The Hospital

An Institutional Journal of

The Medical Sciences and Hospital Administration, with

Vol. XVIII.—No. 453.] AN EXTRA NURSING SUPPLEMENT. [June 1, 1895.

### THE SCHOOLMASTER OR THE DOCTOR?

The attitude of the schoolmaster towards what he considers to be the somewhat aggressive demands of the modern doctor was thus expressed by a successful headmaster the other day: "You have, and are right in having, your professional standpoint. I have, and am right in having, mine." Nevertheless, though he had demurred a good deal, and pleaded strenuously for the maintenance of unbroken school traditions and discipline, he yielded to the doctor's reasoned wishes.

It is hardly possible to imagine a more serious situation than the contest between certain schoolmasters on the one hand and certain modern, deeply-read, and far seeing physiologists on the other. In discussing the subject a man may well ask, like Milton, for an illumination higher than any which comes from human sources, and may—nay must—strive with all his powers to "rise to the height of his great argument," and justify the imperative demands of science to all, whether teachers or parents, boys or girls, who have any personal concern in those demands.

Any man who takes a careful survey of the history of the world and the development of morals and science must be struck by this circumstance, that discoveries of all kinds, in science as in morals, seem to have been made at some peculiarly psychological moment; just, in fact, when the world was needing them, was ripe for them, was imperatively demanding them. By way of examples in science we may mention the discovery of the methods of utilising steam, of the electric telegraph, of anæsthetics; and in morals, the birth and development of Christianity. These observations, we hold, apply with full force to the discoveries of modern physiology and medicine. For the great discoveries of physiology and medicine are almost exclusively modern; and it is the most natural thing in the world for the philosophical inquirer to ask, Why are they modern? Why, in the long history of man, were they never made before? Why did not the early Egyptians, who achieved so much in practical science, investigate and make known the structure and functions of the human body? Why did not Aristotle, who not only took all knowledge for his province, but had, perhaps, the most competent brain for physical investigation the world has ever seen—why did not he discover the ten thousand facts in human and animal physiology, which have since been discovered by men of whom many were intellectual pigmies compared with that great master?

There is one great reason, we hold, why modern physiology and medicine, so long hidden from man, have at last emerged, as from a chrysalis stage of uncounted centuries, to the open day and before all men's eyes. That reason is the dire necessity of the modern world. Never before had the world such tasks set before it as now; never before did it need men of such powerful and enduring physique, such breadth and depth of culture, such courage and resolution to undertake great tasks, and to carry them through with a wide scientific intelligence and an untiring purpose, which knows no satisfaction until they are completed.

If these be the tasks of the world, who is to undertake them? The battle of Waterloo, it is said, was won on the playing fields of Eton. That the conquering of the French at Waterloo demanded extraordinary qualities we do not deny; and that those qualities were found in abundance among Wellington's generals and officers we gladly admit. In like manner we would have other great victories won on the playing fields of Eton and other great schools; victories, not of armies only, but of peace, of science, of statesmanship, of moral and material progress. But if such victories are to be won, the training for them must be as much wider and more scientific than the older training as the tasks of the present day are wider and more scientific than the one supreme task of the early part of the century, the task of defeating Napoleon at Waterloo.

There are three things above all others which are indispensable for the modern man—physique, scientific culture, and character. Those things must be acquired or perfected at school. If they are not, the probability is they will never be acquired or perfected at all. The modern schoolmaster admits the necessity for character absolutely. The necessity for culture he also admits. But when that culture is demanded to be scientific, he does not give his "unfeigned assent and consent": he consents with a grudge. In like manner he admits the necessity for physique; but he insists that it shall be the physique which he has always been accustomed to; the physique of mere muscle, of broad and stalwart shoulders. But the doctor looks deeper: he demands the physiqne of a well-developed, but not over-developed, nervous system; of a trained, but not exhausted, brain; of pure and rich blood; of sound internal organs and well regulated functions. In short, seeing the mighty tasks which the modern man, especially the modern Englishman, has to undertake at home and in every part of the world, he demands that the modern boy shall be so trained in every part as to be a mental, moral, and physical gladiator, prepared for any conflict, of mind, character, and physique, which our own or any other part of the world may challenge him to. The schoolmaster alone cannot give us gladiators of this class. But the schoolmaster, with the doctor, can, and must.

